# Electrospun Heparin-Loaded PCL Nanofiber Sutures with Sustained Release for Antithrombotic Applications

**DOI:** 10.3390/ph19091389

**Published:** 2026-09-02

**Authors:** Yixiang Pan, Yiting Zhu, Wenjie Chen, Jie Xu, Jinhang Ma, Odinaka Cassandra Ezekiel, Hailv Ye, Jiang Wu

**Affiliations:** 1 Key Laboratory of Biotechnology and Pharmaceutical Engineering, School of Pharmaceutical Sciences, Wenzhou Medical University, Wenzhou 325035, China; 2250025786@student.must.edu.mo (Y.P.); yt.zhu3@siat.ac.cn (Y.Z.);; 2 School of Pharmacy and Laboratory of Drug Discovery from Natural Resources and Industrialization, Macau University of Science and Technology, Macau SAR 999078, China; 3 Institute of Biomedicine and Biotechnology, Shenzhen Institutes of Advanced Technology, Chinese Academy of Sciences, Shenzhen 518055, China; 4 Department of Biology, College of Science, Mathematics and Technology, Wenzhou-Kean University, Wenzhou 325060, China

**Keywords:** heparinized surgical sutures, electrospinning, anticoagulant effect, postoperative thrombosis

## Abstract

**Background/Objectives:** Conventional surgical sutures can promote local coagulation after implantation, potentially compromising vascular patency. Heparin is an effective anticoagulant, but systemic administration may cause bleeding and thrombocytopenia. This study aimed to develop an electrospun heparin-loaded polycaprolactone (PCL) suture that combines structural support with sustained local heparin delivery and antithrombotic activity. **Methods:** Heparin was incorporated into electrospun PCL sutures at nominal heparin-to-PCL feed ratios of 0.05 and 0.50 wt%. Fiber morphology, chemical characteristics, tensile properties, heparin release, molecular interactions, cytocompatibility, hemolysis, and plasma recalcification were evaluated. Molecular dynamics simulations were used to investigate PCL–heparin interactions. In vivo antithrombotic performance was assessed in a liver puncture model using male C57BL/6 mice, followed by histological examination. **Results:** Heparin incorporation preserved the fibrous morphology of the sutures, although maximum tensile stress and strain were modestly reduced compared with PCL alone. Both heparin-loaded formulations exhibited sustained heparin release over 10 days, and molecular dynamics simulations indicated stable PCL–heparin interactions. Cell viability did not differ significantly among the suture groups, and all hemolysis rates remained below 5%. In the plasma recalcification assay, both heparin-loaded groups remained incompletely coagulated at 300 s, indicating marked anticoagulant activity. In the mouse liver injury model, heparin-loaded sutures reduced local clot formation compared with PCL sutures. **Conclusions:** Electrospun heparin-loaded PCL sutures integrate sustained local heparin delivery with in vitro anticoagulant and in vivo antithrombotic activity. These findings support further evaluation of this multifunctional suture platform in clinically relevant vascular models.

## 1. Introduction

Vascular suturing and anastomosis are among the most fundamental techniques in the surgical treatment of major arterial and venous diseases or injuries, where blood flow reconstruction often requires direct repair, patch angioplasty, or vascular grafting [1]. The quality of vascular suturing and anastomosis directly influences postoperative vascular patency, with surgical suture material representing a critical determinant [2]. Conventional sutures, however, generally exhibit poor hemocompatibility. Once implanted, they provide surfaces for plasma protein and platelet adsorption, which trigger platelet activation and aggregation, accelerate thrombin generation, and ultimately contribute to postoperative thrombosis [3]. Notably, luminal thrombosis after surgical treatment has become the leading cause of mortality in patients with cardiovascular disease [4].

Heparin (HP) is a naturally occurring, highly sulfated linear glycosaminoglycan [5] with potent anticoagulant activity. It exerts its anticoagulant effects by binding to antithrombin III (ATIII) and accelerating the inactivation of coagulation factors. Consequently, heparin has been widely recognized for its efficacy in the prevention and treatment of deep vein thrombosis [6,7]. Nevertheless, clinical administration of heparin is complicated by considerable interindividual variability in sensitivity, and direct injection frequently leads to adverse effects such as bleeding, thrombocytopenia, and hyperkalemia [8,9]. Surface heparinization of sutures has been explored to enhance bioavailability, improve hemocompatibility, and reduce protein and platelet adhesion. Although promising, such modifications remain limited by rapid release and inactivation of surface-bound heparin, resulting in diminished anticoagulant efficacy over time and reduced clinical utility. Therefore, the development of novel drug-loaded sutures that overcome premature heparin loss and sustain anticoagulant function is urgently needed.

With advances in nanoscience and technology, electrospinning has emerged as a versatile approach for fabricating biomedical materials. Electrospun nanofibrous sutures, composed of aligned nanofiber bundles, offer distinct advantages over conventional sutures, including high surface area for drug loading and the ability to provide sustained drug release [10,11,12]. Recent studies have further demonstrated the potential of electrospun polymeric nanofibers as controlled drug-delivery platforms. For example, PVA/PAA electrospun nanofibers enabled prolonged capecitabine release, while 5-fluorouracil-loaded PVA/PVP nanofibers exhibited high drug loading and controlled release [13,14]. These studies demonstrate that incorporation of therapeutic agents into electrospun polymeric matrices can effectively modulate drug-release behavior. Owing to these advantages, electrospun nanofibrous sutures have been increasingly explored for their regenerative and antibacterial properties [15,16,17,18,19,20,21]. However, their application in anticoagulant functionalization remains insufficiently investigated, particularly in the context of vascular suturing and anastomosis, where long-term hemocompatibility and sustained antithrombotic activity are critically required. PCL, a slow biodegradable and biocompatible polymer, has attracted considerable attention as a promising material, particularly when combined with nanofibrous structures generated via electrospinning [22]. Encapsulating heparin within PCL creates a physical barrier that enables sustained drug release while simultaneously harnessing the favorable mechanical properties of PCL. This strategy improves heparin stability, prolongs its half-life, and enhances anticoagulant efficacy, thereby addressing the burst release and rapid inactivation observed in surface-heparinized sutures. It also improves the overall biocompatibility and mechanical performance of the sutures, further increasing their translational potential.

In this study, we developed heparin-loaded nanofibrous surgical sutures via electrospinning. The microstructure and mechanical properties of the sutures were characterized, and molecular dynamics simulations were employed to confirm stable binding interactions between PCL and heparin, as well as the structural integrity and sustained-release capacity of the nanofibers. Furthermore, we systematically evaluated their biocompatibility, anticoagulant activity, duration of anticoagulant effects, and in vivo performance.

## 2. Results and Discussion

### 2.1. Synthesis and Characterization of Heparinized Antithrombotic Surgical Sutures

**Figure 1:**
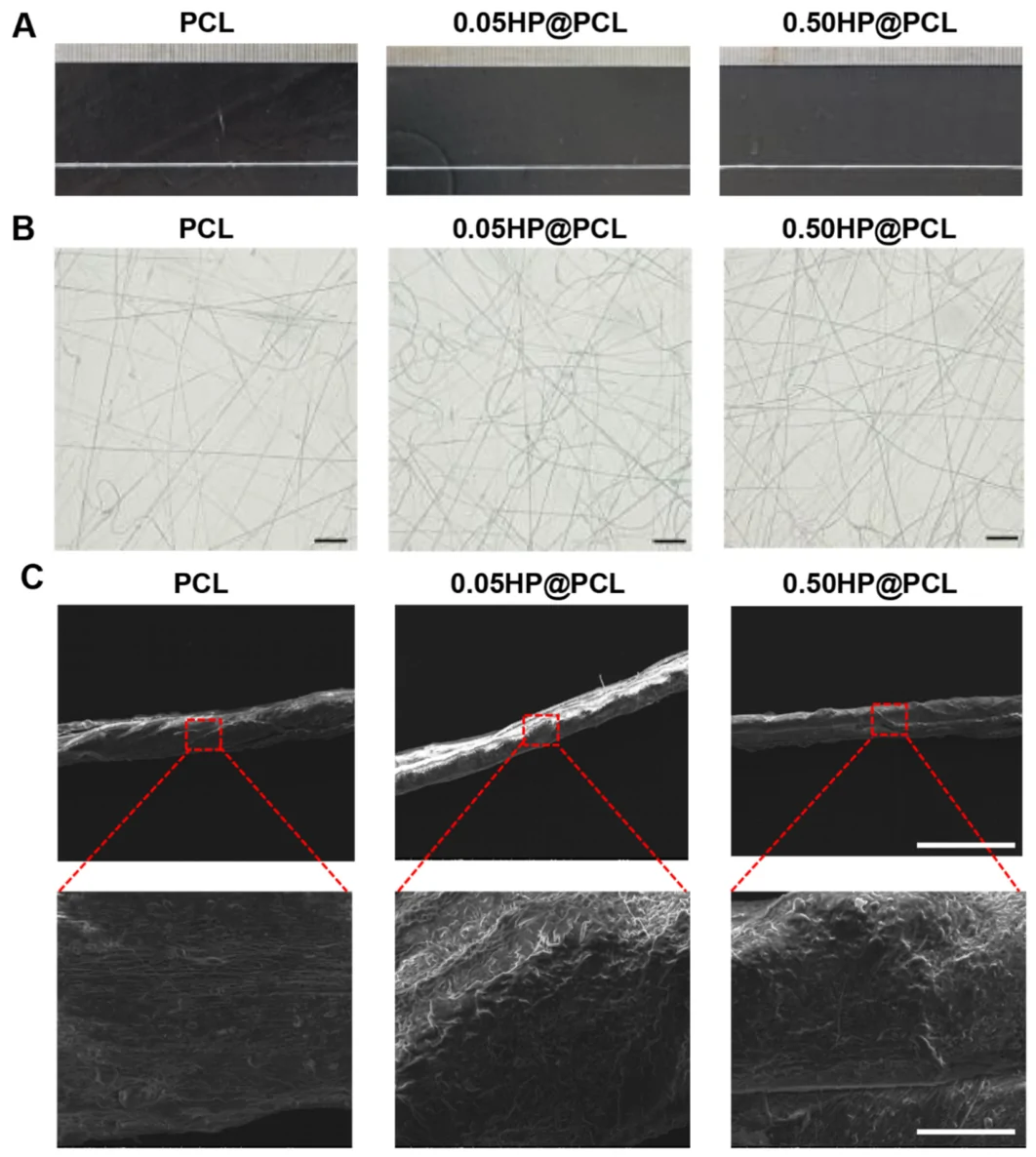
Synthesis and characterization of heparinized antithrombotic surgical sutures. (**A**) Morphology of the electrospun surgical sutures from the PCL, 0.05HP@PCL, and 0.50HP@PCL groups (**B**) Structure of electrospun fibers in the PCL, 0.05HP@PCL, and 0.50HP@PCL groups, under the microscope. Scale bar: 1 mm. (**C**) SEM images of the structure of electrospun fibers in the PCL, 0.05HP@PCL, and 0.50HP@PCL groups. Scale bar: 500 μm (top), 50 μm (bottom).

As shown in Figure 1A, the electrospun nanofiber sutures fabricated under the optimized conditions were continuous and straight, with a uniform diameter. An upright optical microscope was employed to observe and characterize the morphology of electrospun nanofibers (Figure 1B). All three groups of polymer solutions produced continuous, uniform nanofiber bundles, and the presence of heparin did not adversely affect fiber formation. The surface microstructure of the nanofiber sutures was further analyzed using SEM. All solutions produced smooth, uniform nanofiber bundles that aligned in the same direction, forming a thread-like structure with a neat, dense surface arrangement. Compared to pure PCL electrospinning, the addition of heparin did not alter the surface structure of the sutures. This confirms that the drug-loaded nanofiber sutures retain a complete thread-like structure composed of highly oriented, uniform nanofiber bundles, as shown in Figure 1C. Furthermore, due to the poor solubility of heparin sodium in methanol and dichloromethane, a thin white film formed on the outer surface of the drug-loaded sutures following modification of the PCL matrix with heparin.

### 2.2. Heparin Modification Slightly Reduced but Preserved Acceptable Mechanical Properties of the Sutures

**Figure 2:**
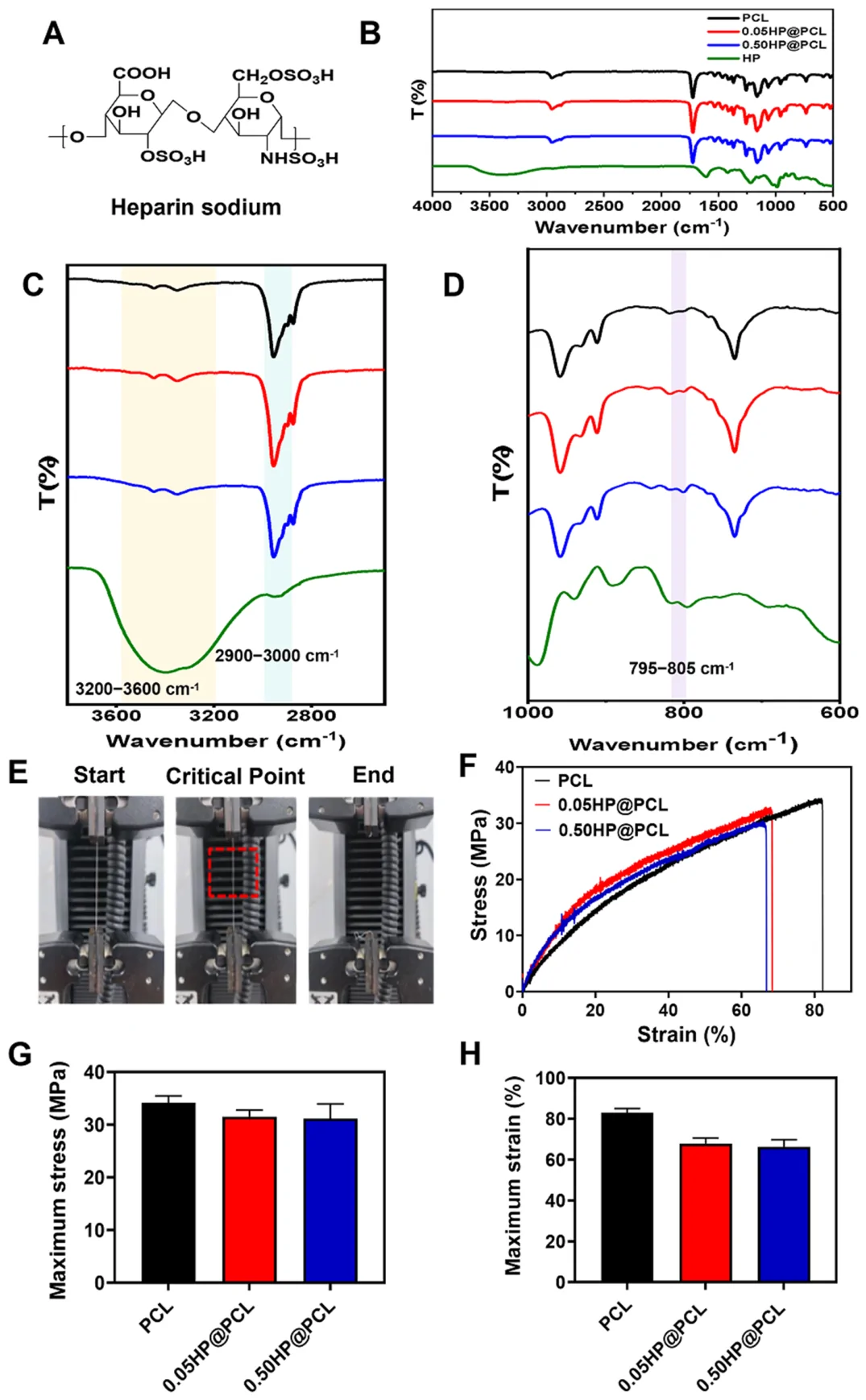
The mechanical property graphs of PCL, 0.05HP@PCL, and 0.50HP@PCL surgical sutures and the ATR−FTIR spectra of their respective sample surfaces were analyzed. (**A**) Molecular structure of heparin. (**B**) Full-range scans (500−4000 cm^−1^) of PCL, 0.05HP@PCL, 0.50HP@PCL, and HP sutures. (**C**) Local magnification (2500−4000 cm^−1^) of PCL, 0.05HP@PCL, 0.50HP@PCL, and HP sutures. (**D**) Local magnification (400−1000 cm^−1^) of PCL, 0.05HP@PCL, 0.50HP@PCL, and HP sutures. (**E**) Single stretch deformation diagram for PCL, 0.05HP@PCL, and 0.50HP@PCL surgical sutures. (**F**) Tensile stress–strain curves for PCL, 0.05HP@PCL, and 0.50HP@PCL surgical sutures. (**G**) Maximum tensile stress diagram for PCL, 0.05HP@PCL, and 0.50HP@PCL surgical sutures. (**H**) Maximum tensile strain diagram for PCL, 0.05HP@PCL, and 0.50HP@PCL surgical sutures. Data in (**G**,**H**) are presented as means ± SDs (*n* = 3 independent experiments per group).

The successful loading of heparin was further confirmed by ATR-FTIR analysis (Figure 2A–D). As shown in Figure 2A, the repeating unit structure of PCL is [CH_2_-(CH_2_)_4_-COO]_n_, while the molecular structure of heparin is a linear polysaccharide composed of repeating units of glucosamine, glucuronic acid, and iduronic acid [23,24]. The characteristic absorption peaks of heparin include the S=O stretching vibration around 1200 cm^−1^, the C-O-C vibration around 1050 cm^−1^, the C-O-S stretching vibration around 800 cm^−1^, the strong carboxylate absorption around 1640 cm^−1^, and the N-H and O-H stretching vibrations between 3000 and 3600 cm^−1^ [25,26]. The absorption peaks of carboxyl, amino, and hydroxyl groups in heparin may interact with the carboxyl and C-H stretching vibration absorption peaks of PCL through overlapping and resonance. Figure 2B shows the ATR-FTIR spectra for the PCL, 0.05HP@PCL, 0.50HP@PCL groups, and the pure HP group within the range of 500–4000 cm^−1^, while Figure 2C,D are local magnifications. From Figure 2C, it can be seen that the pure HP group shows a broad peak (77.33% T) produced by the stretching vibrations of -NH2 and -OH in the range of 3000–3600 cm^−1^. The 0.50HP@PCL group (96.64% T) and the 0.05HP@PCL group (98.50% T) also show broad peaks in this range, while the PCL group shows no peak in this region. Additionally, Figure 2D shows that the pure HP group exhibits a distinct C-O-S stretching vibration at 795 cm^−1^. The 0.05HP@PCL and 0.50HP@PCL groups also show absorption peaks in this position, and the intensity of the absorption peaks increases with the concentration of heparin, whereas the PCL group does not show a peak. The remaining characteristic peaks of heparin were not clearly resolved, most likely due to overlap with the absorption bands of PCL. Nevertheless, the presence of the broad O–H/N–H stretching band at 3000–3600 cm^−1^ and the C-O-S stretching band at 795 cm^−1^ provided evidence for the successful incorporation of heparin into the sutures.

Surgical sutures prepared with PCL as the matrix material inherently possess excellent mechanical properties. To identify variations in the mechanical properties of heparin-loaded sutures, a universal testing machine was utilized to perform a single tensile test. Figure 2E shows the changes in the sutures before and after stretching. All three groups of sutures underwent some deformation following stretching. Tensile stress increased with tensile strain, and the material broke when the critical stress was reached. As shown in Figure 2F, heparin incorporation reduced the tensile strength and elongation. This reduction may be associated with heparin-induced changes in fiber morphology and the internal organization of the PCL matrix. Consistent with our morphological observations, a previous study reported that increasing the heparin content reduced the diameter of electrospun PCL fibers, potentially due to increased ionic charge in the spinning solution [27]. As summarized in Figure 2G,H and Table 1, heparin incorporation reduced both the maximum tensile stress and strain. No significant difference was observed between the 0.05HP@PCL and 0.50HP@PCL groups (*p* > 0.05), indicating that the reduction was independent of heparin loading.

Although the heparin-loaded sutures retained favorable tensile properties, direct comparison with pharmacopoeial requirements was not included in the present study. Future studies should further evaluate knot-pull strength and breaking load in accordance with relevant surgical suture standards.

### 2.3. Drug Release Behavior and Underlying Molecular Mechanisms

To assess the sustained-release performance of heparin from the surgical sutures, in vitro release experiments were performed. The release profiles were fitted using zero-order, first-order, Higuchi, and Korsmeyer–Peppas models, and the goodness of fit was evaluated using the coefficient of determination (R2), as shown in Table 2. Among these models, the Korsmeyer–Peppas model provided the best fit for the experimental release data and was therefore selected to further characterize the heparin release mechanism. The representative fitted curves are presented in Supplementary Figures S1 and S2.

**Figure 3:**
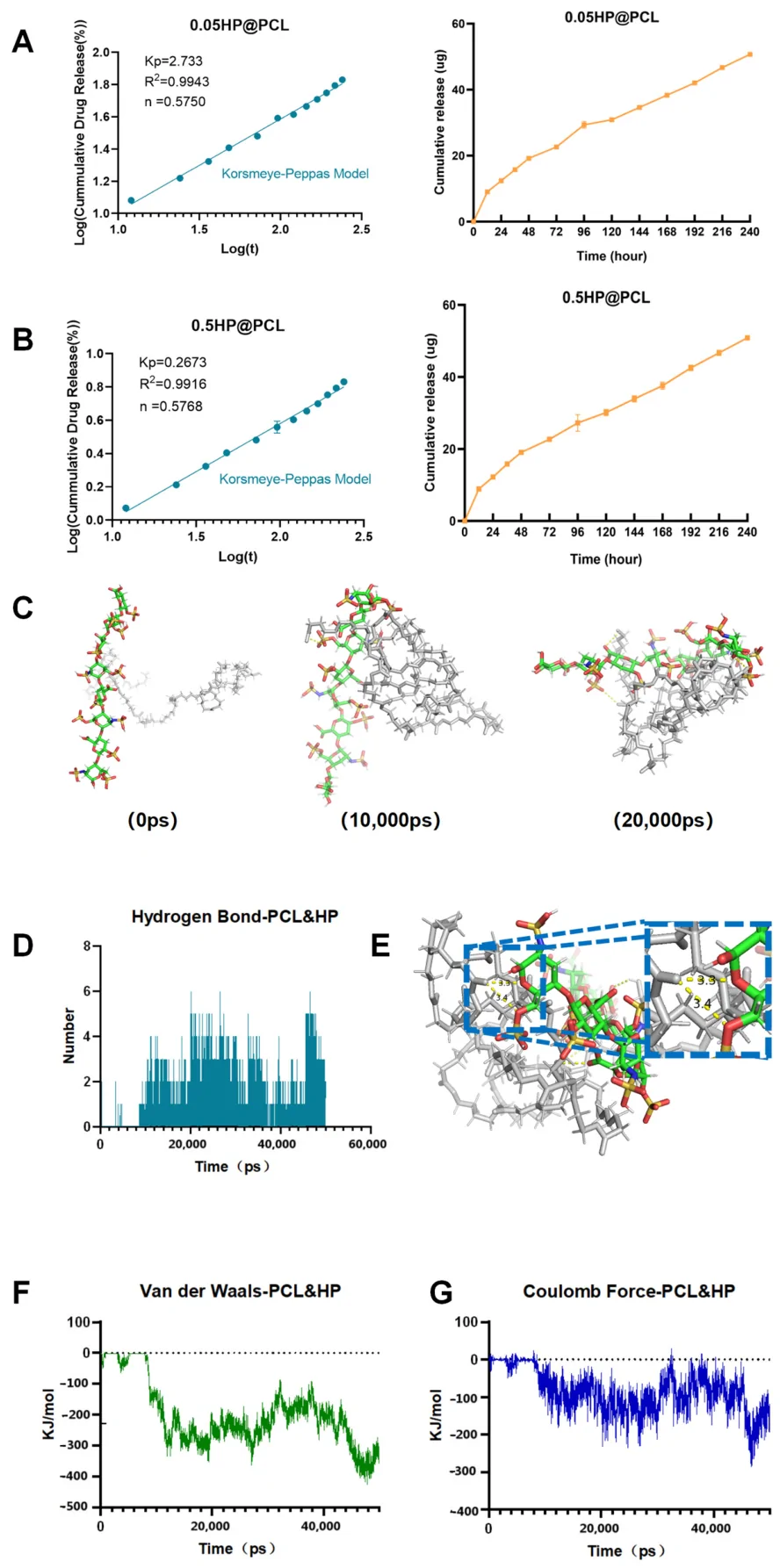
Cumulative heparin release profiles and interaction analysis between PCL and HP. (**A**) 10−day heparin release profile of the 0.05HP@PCL surgical suture: release profile plotted against log-transformed time [Log(t)]; cumulative heparin release plotted against actual time (h). (**B**) 10−day heparin release profile of the 0.50HP@PCL surgical suture: release profile plotted against log-transformed time [Log(t)]; cumulative heparin release plotted against actual time (h). (**C**) Binding of the PCL chain and the HP molecule, showing the unbound state (0 ps), contact state (10 ns), and fully contacted state (20 ns). (**D**) Number of hydrogen bonds formed between PCL and heparin over a 50 ns simulation. (**E**) Visualization of hydrogen bond formation between PCL and heparin. In (**C**,**E**), C, O, S, and N atoms are represented by green, red, yellow, and blue, respectively. (**F**) Variation curve of van der Waals forces. (**G**) Plot of Coulombic interactions. Release data are presented as means ± SDs from four independent suture samples per formulation (*n* = 4).

As shown in Figure 3A,B, the heparin sodium-modified PCL sutures exhibited stable and sustained release profiles in PBS. During the 240 h observation period, the cumulative heparin release gradually increased, indicating that the PCL-based suture system enabled controlled heparin release. Notably, although heparin sodium is intrinsically unstable and prone to degradation, its biological activity remained detectable throughout the release period, suggesting that encapsulation within the PCL matrix effectively preserved its bioactivity. Both 0.05HP@PCL and 0.50HP@PCL sutures exhibited similar cumulative release trends, which may be attributed to the hydrophobic nature of PCL and the hydrophilic characteristics of heparin sodium. During electrospinning, intermolecular interactions between PCL and heparin sodium may have contributed to the formation of a relatively stable fibrous structure, thereby restricting the rapid diffusion of inner-layer heparin into PBS and enabling slow, sustained release. The release exponent (*n*) values were 0.5750 and 0.5768 for the 0.05HP@PCL and 0.50HP@PCL groups, respectively. As these values fall within the range of 0.45–0.89, the release behavior can be interpreted as anomalous transport involving both diffusion and polymer erosion, with diffusion playing a dominant role [28]. These findings highlight the advantage of PCL as a drug carrier for sustained heparin release, effectively reducing burst release and extending the duration of anticoagulant activity.

Comparison of the complete fitting results further showed that the Korsmeyer–Peppas model yielded the highest R^2^ values for both 0.05HP@PCL and 0.50HP@PCL sutures (0.9943 and 0.9916, respectively), although the zero-order and Higuchi models also provided strong fits. The nearly identical release exponents (*n* = 0.5750 and 0.5768) indicate that increasing the heparin loading did not substantially alter the underlying transport mechanism. This similarity is consistent with the SEM observations showing that both drug-loaded sutures retained densely packed, highly oriented electrospun fiber bundles. Such a fibrous PCL matrix can restrict water penetration and increase the diffusion path for heparin, while the intermolecular interactions between PCL and heparin further retard drug migration; together, these structural features support a diffusion-dominated anomalous release process coupled with limited matrix relaxation or erosion.

To further elucidate the molecular basis of sustained release, molecular dynamics (MD) simulations were conducted to examine interactions between PCL and heparin (HP). Over the 50 ns simulation, PCL chains rapidly aggregated in water, whereas HP molecules initially remained relatively free in solution. After approximately 10 ns, HP began to contact and progressively entangle with PCL, eventually forming a stable complex (Figure 3C). Hydrogen bond analysis revealed that two to three stable hydrogen bonds were formed between PCL and HP during the periods of 10–30 ns and 45–50 ns (Figure 3D). Specifically, the carbonyl oxygen (C=O) in PCL acted as a hydrogen bond acceptor, interacting with hydroxyl (−OH) or amino (−NH_2_) groups in heparin. The terminal hydroxyl groups (−OH) of PCL could serve as either hydrogen-bond donors or acceptors, forming bonds with sulfonate (−SO_3_^−^) or hydroxyl groups in heparin (Figure 3E). These hydrogen bonds significantly enhanced the interaction between PCL and HP, thereby improving the stability and biocompatibility of the material.

Furthermore, both PCL and HP contain polar groups (e.g., carbonyl, hydroxyl, and sulfonate), which cause uneven electron distribution, induce transient polarization, and generate van der Waals forces. As shown in Figure 3F, the Lennard–Jones potential indicated van der Waals interaction energies ranging from −200 to −300 kJ/mol after 1 ns of simulation. Simultaneously, electrostatic interactions played a crucial role in the binding process: the partially negatively charged carbonyl oxygen in PCL attracted the positively charged amino groups (−NH_3_^+^) in heparin, and the negatively charged sulfonate groups in heparin could interact electrostatically with slightly positive impurities or protonated carbonyl groups in PCL (Figure 3G). The Coulombic interaction energy was approximately −100 to −150 kJ/mol.

Overall, the electrospun PCL fibrous matrix provides structural support while serving as a reservoir for heparin incorporated within the fibers during fabrication. Upon exposure to an aqueous environment, the incorporated heparin is gradually released from the PCL matrix and subsequently exerts its anticoagulant activity.

Unlike the strategy of heparin immobilization via electrostatic adsorption with cationic polymers [29], this study achieved stable heparin retention through physical encapsulation and intermolecular interactions, thereby avoiding burst drug release and improving the stability of long-term anticoagulation. In summary, the molecular dynamics simulations show that PCL and heparin bind stably through hydrogen bonding, van der Waals forces, and electrostatic interactions, providing a mechanistic explanation for the sustained heparin release observed experimentally.

### 2.4. Heparinized Antithrombotic Surgical Sutures Exhibit Good In Vitro Cytocompatibility

**Figure 4:**
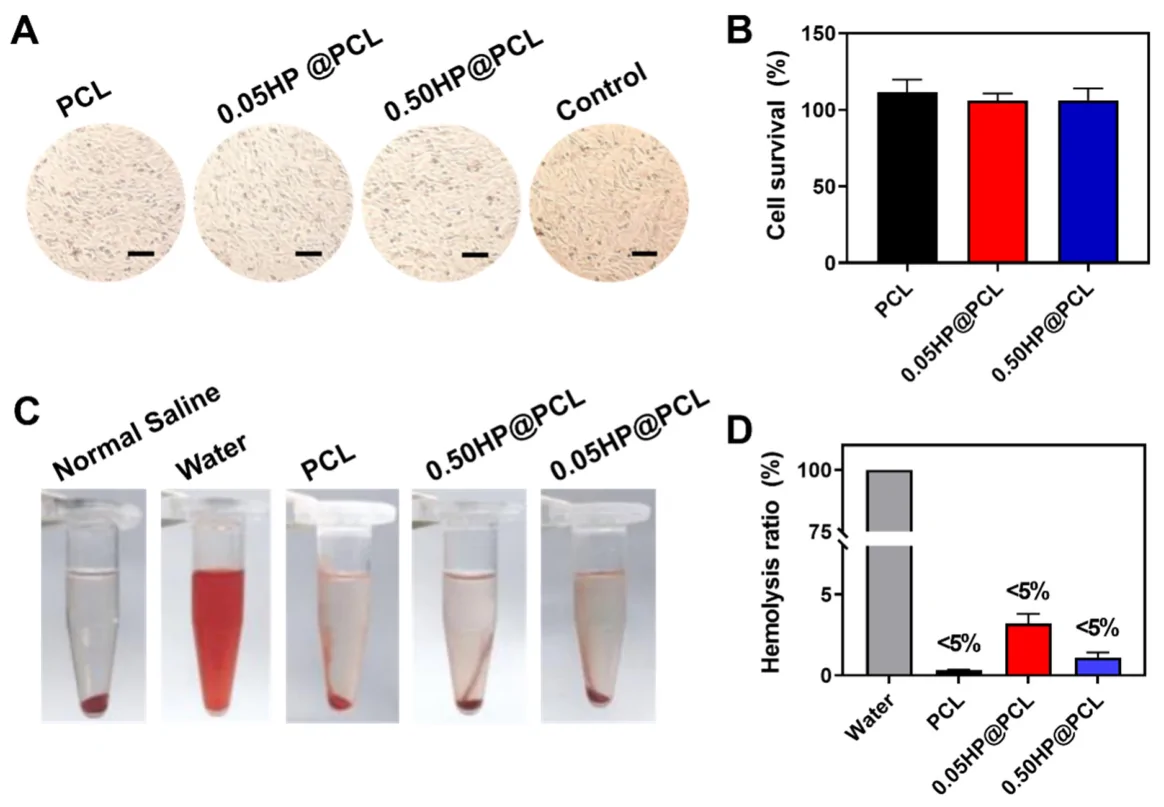
In vitro cytotoxicity assay and hemolysis test of the heparinized antithrombotic surgical sutures. (**A**) Cytocompatibility assessment of the surgical sutures. Representative images of fibroblast morphology after incubation with PCL, 0.05HP@PCL, and 0.50HP@PCL sutures, compared with the Control group. Scale bar: 40 μm. (**B**) In vitro MTT assay of the PCL, 0.05HP@PCL, and 0.50HP@PCL groups (*n* = 5 independent suture extracts per group). (**C**) Hemolysis of blood cells following centrifugation with PCL, 0.05HP@PCL, and 0.50HP@PCL. (**D**) In vitro hemolysis rate test for each group (*n* = 3 independent suture samples per group).

Figure 4A shows the cell morphology after overnight incubation with extracts from the three surgical sutures and pure water as a control. It can be observed that cells in all four groups grew well, with regular morphology and similar density. Figure 4B indicates that cell viability in the PCL, 0.05HP@PCL, and 0.50HP@PCL groups exhibited a higher level, while no statistically significant differences were observed among these groups (*p* > 0.05). This suggests that the sutures in all groups are non-cytotoxic and possess a high degree of biocompatibility.

The hemolysis rate reflects the extent of harm biomaterials cause to blood cells; a lower hemolysis rate indicates a reduced risk of thrombus formation and improved blood compatibility. According to the international standard ISO 10993, the hemolysis rate of materials should be below the safety threshold of 5% [30].

Figure 4C illustrates the appearance of blood cell suspensions in centrifuge tubes after incubation and centrifugation. Physiological saline, used as the negative control, showed no hemolysis due to its isotonicity, with all blood cells settling at the bottom of the tube. Similarly, the three groups exhibited substantial red blood cell deposition without evident hemolysis. The corresponding in vitro hemolysis rates are shown in Figure 4D, with values of 0.31 ± 0.05%, 3.19 ± 0.62%, and 1.09 ± 0.32% for the three suture groups, respectively. All values were below 5%, thus meeting international safety standards.

The experimental results confirmed that the prepared PCL, 0.05HP@PCL, and 0.50HP@PCL surgical sutures exhibited no cytotoxicity. However, despite the favorable in vitro biocompatibility observed in this study, potential heparin-related risks should be considered. A fraction of locally released heparin may enter the systemic circulation, increasing the risk of bleeding or heparin-induced thrombocytopenia. Future studies should therefore evaluate systemic heparin exposure, coagulation parameters, platelet counts, and local bleeding complications to further establish the safety of these sutures.

### 2.5. In Vitro Anticoagulant Activity

**Figure 5:**
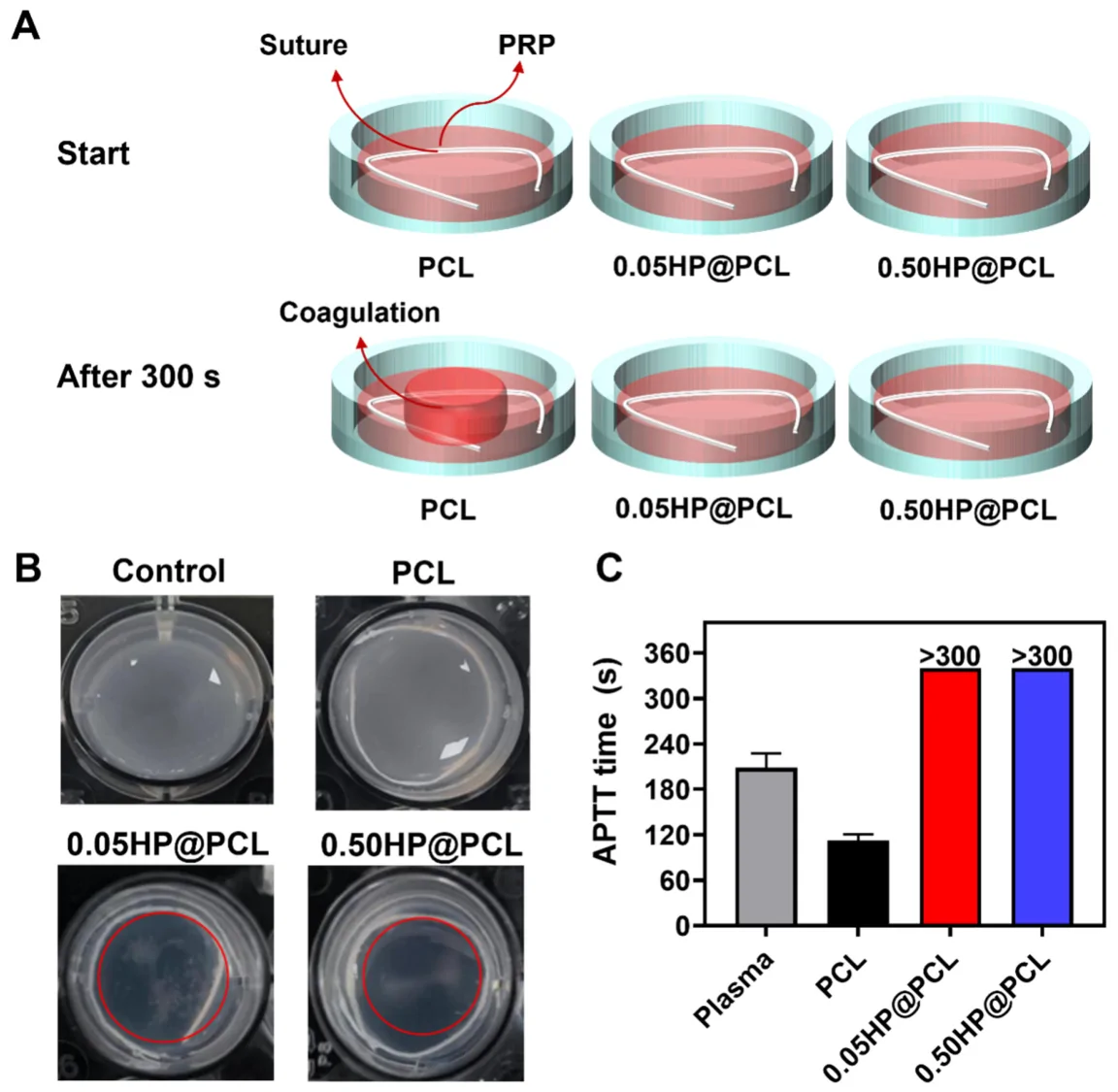
In vitro anticoagulant activity of heparinized antithrombotic surgical sutures. (**A**) Schematic diagram of the in vitro anticoagulation experiment for PCL, 0.05HP@PCL, and 0.50HP@PCL surgical sutures. (**B**) Representative images of the in vitro anticoagulation experiment for PCL, 0.05HP@PCL, and 0.50HP@PCL surgical sutures. (**C**) Coagulation time statistics for the plasma, PCL, 0.05HP@PCL, and 0.50HP@PCL groups. Data are presented as means ± SDs from three independent experiments per group (*n* = 3).

As illustrated in Figure 5A (schematic) and Figure 5B (experimental images), both 0.05HP@PCL and 0.50HP@PCL sutures exhibited markedly greater anticoagulant activity than the PCL and plasma control groups. Compared to the PCL group (112.5 ± 8.08 s) and the pure plasma group (208.6 ± 18.86 s), even when triggered by CaCl_2_, the addition of heparin-loaded sutures did not fully clot the bovine PRP. HP@PCL groups showed only initial coagulation with turbid precipitation, indicating that the addition of heparin successfully delayed and prevented activation of the intrinsic coagulation pathway. As shown in Figure 5B, both the 0.05HP@PCL and 0.50HP@PCL sutures exhibited significant anticoagulant effects compared with the control group. Consistently, the quantitative analysis in Figure 5C showed that neither group achieved complete coagulation within the 300-s observation period. Because both groups exceeded this observation limit, their anticoagulant capacities could not be quantitatively distinguished based on clotting time alone. Therefore, we further evaluated the antithrombotic performance of the sutures in vivo.

### 2.6. In Vivo Antithrombotic Performance

**Figure 6:**
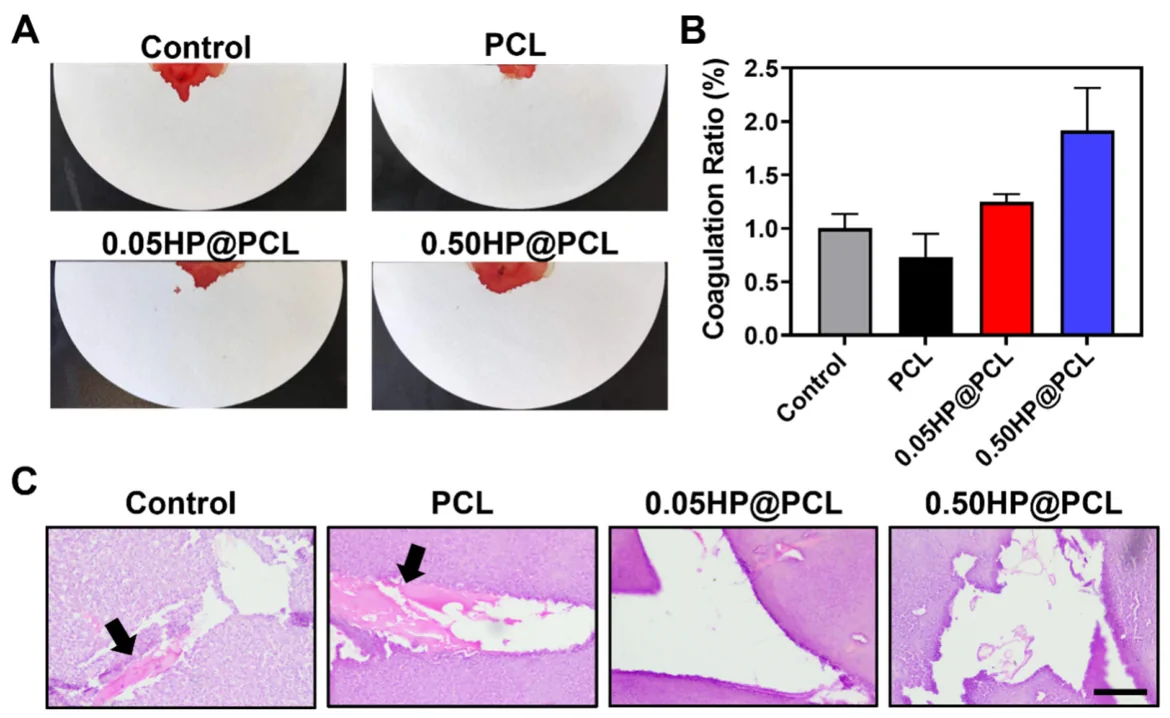
In vivo mouse liver injury results for the heparinized antithrombotic surgical sutures. (**A**) Representative images of blood absorption in the Control, PCL, 0.05HP@PCL, and 0.50HP@PCL groups. (**B**) Bar chart showing the amount of liver blood loss in the control, PCL, 0.05HP@PCL, and 0.50HP@PCL groups. (**C**) H&E staining images of liver tissues from the Control, PCL, 0.05HP@PCL, and 0.50HP@PCL groups. Black arrows indicate areas of thrombus formation. Scale bar: 200 μm.

Building on the favorable in vitro anticoagulant performance, we further evaluated whether the heparin-modified sutures could exert effective antithrombotic activity under physiological conditions in vivo. A mouse liver puncture model was established because the liver is highly vascularized, enabling direct evaluation of the local anticoagulant effects of the sutures. As shown in Figure 6A, the PCL group exhibited the smallest blood-stained area, indicating improved hemostasis after suturing. This hemostatic effect may also be associated with the adsorption of plasma proteins and platelets onto the PCL surface, which promotes clot formation [31]. In contrast, the blood-stained area increased with heparin loading and was greatest in the 0.50HP@PCL group. Consistently, the coagulation ratio was lower in the PCL group than in the control group but increased progressively in the heparin-loaded groups (Figure 6B). These results indicate that heparin loading inhibited local clot formation in a dose-dependent manner, confirming the anticoagulant activity of the HP@PCL sutures.

To further investigate the in vivo antithrombotic performance of the sutures, H&E staining was performed on livers. As shown in Figure 6C, obvious thrombi (marked by black arrows) were detected in both the control group and the PCL group, among which the thrombi formed in the PCL group were larger in size than those in the control group. However, in the 0.05HP@PCL group and the 0.50HP@PCL group, blood clots were virtually non-existent. The results indicate that, compared with the control groups, heparin-modified sutures exhibited stronger inhibition of blood clot formation and enhanced antithrombotic activity. This effect may be attributed to the release of heparin, which further prevents their aggregation.

To place these findings in the context of previous electrospun suture systems, several studies have demonstrated that electrospun nanofiber yarns can integrate the structural function of a suture with localized therapeutic delivery. For example, drug-eluting electrospun nanofiber yarns have been developed for postoperative wound healing, in which the fibrous architecture simultaneously provided mechanical support and sustained local drug delivery [16,32]. In the field of anticoagulant sutures, Bae et al. developed PLGA/PEO/PgP heparin-eluting electrospun nanofiber yarns that achieved sustained heparin release and prolonged coagulation time in vitro [29]. However, their antithrombotic performance was not evaluated in vivo. Other electrospun suture systems, such as PLLA/PA6 core–shell sutures, have mainly been developed for tissue repair rather than anticoagulant applications [19]. In comparison, the present design directly incorporates heparin within the electrospun PCL fibrous matrix, allowing the suture itself to serve as both a structural material and a local drug-delivery reservoir. Compared with the previously reported PLGA/PEO/PgP heparin-eluting system, the present PCL/heparin suture uses a compositionally simpler matrix and extends the evaluation from in vitro anticoagulant characterization to proof-of-concept in vivo antithrombotic assessment. Moreover, molecular dynamics simulations were incorporated to provide mechanistic insight into the interactions between PCL and heparin. Thus, the significance of this strategy lies not simply in the PCL/heparin combination, but in transforming a passive suture into a multifunctional therapeutic platform that integrates tissue approximation, sustained local heparin delivery, and local antithrombotic activity.

Despite these encouraging findings, the mouse liver injury model does not recapitulate the hemodynamic environment of arterial or venous anastomosis, including continuous blood flow, vascular shear stress, and the vascular endothelial environment. Therefore, the present animal experiment should be regarded as proof of concept for local antithrombotic activity rather than direct validation of vascular anastomosis performance. Future studies using vascular anastomosis or vascular graft models are required to further evaluate the efficacy and safety of these sutures under clinically relevant blood-flow conditions.

## 3. Materials and Methods

### 3.1. Materials

Heparin sodium salt (HP), characterized by an activity of 185 USP units per milligram, was sourced from Shanghai Macklin Reagent (Shanghai, China). Additionally, polycaprolactone (PCL) with an average molecular weight of 80,000 was procured from Sigma-Aldrich (St. Louis, MO, USA). Methanol was obtained from Changshu Hongsheng Fine Chemical (Changshu, China). Dichloromethane was obtained from Shanghai Titan Technology (Shanghai, China). Sinopharm Chemical Reagent Co., Ltd. (Shanghai, China) supplied the saline solution. Glass slides were obtained from Sigma-Aldrich (St. Louis, MO, USA). Guangzhou Huada Chemical Reagent (Guangzhou, China) supplied the dimethyl sulfoxide (DMSO). Fresh bovine anticoagulated whole blood treated with sodium citrate was obtained from Nanjing Maojie Microbial Technology (Nanjing, China).

### 3.2. Methods

#### 3.2.1. Fabrication of Heparin-Loaded Suture via Electrospinning

Heparin (0.4 mg, corresponding to a nominal heparin-to-PCL feed ratio of 0.05 wt%, or 4 mg, corresponding to 0.50 wt%) was first dissolved in 150 μL of distilled water. These values represent the initial amounts of heparin added relative to the mass of PCL and do not reflect experimentally determined heparin loading or encapsulation efficiency. Subsequently, 3 mL of methanol and 7 mL of dichloromethane were sequentially added to the solution. PCL (800 mg) was then added and completely dissolved. The resulting solution was loaded into a 10 mL syringe fitted with a 22 G needle and electrospun using a YFSP-GIII electrospinning apparatus(Tianjin Yunfan Technology Co., Ltd., Tianjin, China) under the following conditions: a needle-to-collector distance of 15 cm, a collector rotational speed of 300 r/min, an applied voltage of 15 kV, an infusion rate of 0.0034 mm/s, and a spinning time of 30 min. Uniform electrospun fibers were collected on the rotating collector.

#### 3.2.2. Morphological Characterization of Electrospun Fibers

Electrospun fibers were collected on glass slides for morphological observation under an upright microscope(Nikon Corporation, Tokyo, Japan). Scanning electron microscopy (SEM, Hitachi H-7500, Hitachi, Ltd., Tokyo, Japan) was utilized to analyze the surface structures of the PCL, 0.05HP@PCL, and 0.50HP@PCL sutures.

#### 3.2.3. Mechanical Property Testing of Sutures

The mechanical properties of the three sutures prepared under identical conditions were evaluated. Then, 5 cm sections were tested using a universal testing machine (Instron 3344, Instron, Norwood, MA, USA) to determine the maximum stress, maximum strain, and stress–strain curves. The effects of heparin incorporation on suture toughness and elasticity were assessed based on these results.

#### 3.2.4. ATR-FTIR Characterization

The incorporation of heparin on the suture surface was analyzed by ATR-FTIR using an infrared spectrometer (PerkinElmer, Shelton, CT, USA). Spectra were collected over the 500–4000 cm^−1^ range, and characteristic heparin absorption bands were used to verify successful loading onto the surgical sutures.

#### 3.2.5. Molecular Dynamics Simulation Methods

Molecular dynamics simulations were performed using GROMACS 2021 [33] for 50 ns to verify the binding interactions between PCL and heparin. The degree of polymerization of PCL used in the molecular dynamics simulation was 20, whereas heparin consisted of 8 saccharide units. The structures were drawn and optimized using ChemDraw software 2021.

The CHARMM 36 [34] force field was employed for the system, and the compound parameters were generated using the online tool CGenFF 4.0 [35] based on the CHARMM force field. The PCL compound with a degree of polymerization of 20 and the heparin molecules were placed in a cubic box with a side length of 6 nm, maintaining a 1 nm distance from the box edges. The system was solvated with TIP3P [36] water molecules, and the system charge was neutralized by adding Na^+^ ions to maintain an electrostatically neutral environment.

The molecular dynamics simulation process included several steps: preparation of the model and ligand compounds, addition of ions, energy minimization, equilibration of the system with restrained ligands, production simulation, and result analysis. The system was simulated for 50 ns at a target temperature of 300 K [37] and a target pressure of 1 bar [38]. The trajectory data from the molecular dynamics simulation were analyzed using the trjconv module of Gromacs 2021. The energy, temperature, and pressure changes during the simulation were calculated using the energy module of Gromacs 2021. The root-mean-square deviation (RMSD) values of the system components were computed using the rms module of Gromacs 2021.

#### 3.2.6. Release Profile of Heparin from HP@PCL

The in vitro release of heparin from the electrospun sutures was determined using a toluidine blue colorimetric assay. Heparin sodium standards (1–5 μg/mL) prepared in PBS (pH 7.4) were used to generate a calibration curve. Furthermore, 30-cm-long heparin-loaded sutures were incubated in 3 mL of PBS at 37 °C. At predetermined time points (ranging from 12 to 240 h), 1 mL aliquots were collected and replaced with fresh medium. Toluidine blue was first added to the samples to initiate the reaction, followed by extraction of the reaction mixture with n-hexane. The absorbance of the aqueous phase was then measured at 630 nm after the extraction was complete. Heparin concentration was calculated from the calibration curve, and the cumulative release was determined after applying corrections for serial sampling.

#### 3.2.7. In Vitro Biocompatibility

##### Methylthiazolyl Tetrazolium (MTT)

Cytocompatibility was assessed using an MTT assay. The three groups of sutures were sterilized by overnight UV irradiation, and their extracts were prepared in DMEM at 10 mg/mL for 24 h at 37 ± 2 °C [39]. The mouse fibroblast cell line NIH/3T3 was purchased from Shanghai Zhong Qiao Xin Zhou Biotechnology Co., Ltd. (Shanghai, China). NIH/3T3 cells were seeded in 96-well plates at 1 × 10^4^ cells per well and cultured for 24 h, then incubated with the corresponding suture extracts for another 24 h. Cells cultured in standard medium were used as the control. MTT was then added to a final concentration of 1 mg/mL and incubated for 4 h. After dissolving the formazan crystals in DMSO, absorbance was measured at 490 nm, and cell viability was calculated relative to the control group.

##### Hemolysis Test

The hemolysis assay was performed according to a previously reported method, with minor modifications [40]. Bovine blood anticoagulated with sodium citrate was collected, and the blood cells were washed with saline by centrifugation at 3000 rpm for 5 min until the supernatant was clear. A 5% (*v*/*v*) red blood cell (RBC) suspension was then prepared. A 1.5 cm segment of suture was placed in a tube, dispersed in 500 μL of saline by vortexing, and mixed with 500 μL of the RBC suspension. The mixture was incubated at 37 °C for 30 min and centrifuged at 3000 rpm for 5 min. Then, 100 μL of the supernatant was transferred to a new plate, and the absorbance was measured at 540 nm using a microplate readerSpectraMax 190 microplate reader (Molecular Devices, LLC, San Jose, CA, USA). The hemolysis rate (HR) was calculated using the following formula:

(1) $$\mathrm{HR}\left(\%\right)=\frac{A_{1}-A_{2}}{A_{3}-A_{2}}\times 100\%$$

where *A*_1_, *A*_2_, and *A*_3_ correspond to the absorbance values of the experimental group, the negative control group, and the positive control group, respectively.

#### 3.2.8. Plasma Recalcification Clotting Time Assay

To assess the anticoagulant activity, a plasma recalcification clotting time assay was employed. The experimental procedure references a previously reported method, with minor modifications made to accommodate the current experimental conditions [29]. Bovine whole blood anticoagulated with sodium citrate was subjected to centrifugation. The centrifugation was performed at 3000 rpm for 15 min to obtain platelet-rich plasma (PRP). Suture samples (control, PCL, 0.05HP@PCL, and 0.50HP@PCL) were incubated with 500 μL PRP in 500 μL calcium- and magnesium-free PBS in a sterile 24-well plate. After gentle shaking at room temperature for 5 min, the system was incubated at 37 °C for an additional 20 min, and coagulation was triggered by adding 100 μL of preheated 0.1 M CaCl_2_. Clotting time was determined by visual observation of clot formation, and the average value was calculated for each group.

#### 3.2.9. In Vivo Antithrombotic Evaluation

##### Blood Loss Measurement in a Mouse Liver Injury Model

The in vivo antithrombotic performance of the sutures was evaluated using a mouse liver puncture model. Male C57BL/6 mice (20–25 g) were purchased from Charles River Laboratories (Beijing, China). The animal experiments were conducted at the Laboratory Animals Center of Wenzhou Medical University, with mice randomly assigned to four groups: control, PCL, 0.05HP@PCL, and 0.50HP@PCL. All procedures were reviewed and approved by the Experimental Animal Ethics Committee of Wenzhou Medical University (approval No. wydw2025-0539) and were conducted in accordance with international ethical guidelines and the National Institutes of Health Guide for the Care and Use of Laboratory Animals. Liver puncture was performed using a △1/2 5 × 12 medical suture needle either without modified suture material (control) or with the corresponding loaded sutures. After the needle passed through the liver, the suture was left in place. Bleeding from the wound was immediately collected using pre-weighed filter paper. After 2 min, the filter paper was retrieved and weighed again, and blood loss was determined from the increase in weight. The anticoagulation percentage was calculated relative to the control group. Five independent experiments were performed for each group. The anticoagulation percentage was calculated according to the following formula.

(2) $$A\%=\frac{M_{x}-M_{y}}{M_{1}-M_{0}}\times 100\%$$

where *M_x_* represents the initial weight of the filter paper in the experimental group; *M_y_* represents the final weight of the filter paper in the experimental group; *M*_1_ represents the initial weight of the filter paper in the control group; *M*_0_ represents the final weight of the filter paper in the control group.

##### H&E Staining of Mouse Liver Tissue

Hematoxylin and eosin (H&E) staining was performed on the treated liver wounds to assess local clot formation and further evaluate the in vivo antithrombotic performance of the sutures. After sampling, the liver tissue was fixed in 4% paraformaldehyde for 24 h, dehydrated, embedded in paraffin, and sectioned using a microtome (LEICA RM2235, Leica, Wetzlar, Germany). The sections were stained with hematoxylin and eosin (H&E), and the stained liver wound areas were examined under a microscope to assess the presence or absence of blood clot formation.

#### 3.2.10. Statistical Analysis

All quantitative data are presented as means ± standard deviations (SDs). Statistical analyses were conducted using GraphPad Prism 5 software (GraphPad Software Inc., La Jolla, CA, USA). Differences among multiple groups were evaluated by one-way analysis of variance (ANOVA), followed by Tukey’s post hoc test. Statistical significance was defined as *p* < 0.05, with *p* < 0.01 and *p* < 0.001 indicating greater significance.

## 4. Conclusions

In the present work, a new type of heparin-modified antithrombotic surgical suture was fabricated via electrospinning. Comprehensive characterization of these three sutures showed that heparin incorporation preserved a uniform fibrous morphology, maintained suitable mechanical performance, and enabled sustained and stable heparin release, likely through intermolecular interactions between PCL and heparin. Biological evaluation further demonstrated that the heparinized sutures exhibited good cytocompatibility, hemolysis rates below the accepted safety threshold, and dose-dependent anticoagulant activity, with higher heparin loading producing a stronger effect. In addition, the sutures exhibited effective in vivo antithrombotic performance in a mouse liver injury model. Overall, these findings indicate that electrospun heparinized sutures are a promising strategy for developing multifunctional surgical materials with local anticoagulant activity. Further studies are warranted to evaluate their long-term biocompatibility, degradation behavior, and performance across different surgical settings, thereby providing stronger support for future clinical translation.

## Figures and Tables

**Table 1:** Comparison of Maximum Tensile Stress and Strain for the Three Groups of Sutures.

Group	Maximum Stress (MPa)	Maximum Strain (%)
PCL	34.164 ± 1.279	83.085 ± 1.940
0.05HP@PCL	31.494 ± 1.248	67.803 ± 2.781
0.50HP@PCL	31.133 ± 2.776	66.280 ± 3.520

**Table 2:** The R^2^ values and Model parameters for the 0.05HP@PCL and 0.5HP@PCL groups evaluated using four different models.

Sample Group	Kinetic Model	R^2^	Model Parameter(s)
0.05HP@PCL	Zero-order	0.9887	*K*_0_ = 0.2323
0.05HP@PCL	First-order	0.9019	*K*_1_ = 0.002900
0.05HP@PCL	Higuchi	0.9883	*K*_H_ = 4.523
0.05HP@PCL	Korsmeyer-Peppas	0.9943	*K*_KP_ = 2.733; *n* = 0.5750
0.50HP@PCL	Zero-order	0.9904	*K*_0_ = 0.02334
0.50HP@PCL	First-order	0.9121	*K*_1_ = 0.002926
0.50HP@PCL	Higuchi	0.9850	*K*_H_ = 0.4525
0.50HP@PCL	Korsmeyer-Peppas	0.9916	*K*_KP_ = 0.2673; *n* = 0.5768

## Data Availability

The original contributions presented in this study are included in the article/Supplementary Materials. Further inquiries can be directed to the corresponding authors.

## Supplementary Materials

The following supporting information can be downloaded at: https://www.mdpi.com/article/10.3390/ph19091389/s1, Figure S1: 0.5HP@PCL using four different models to evaluate the release profile and mechanism; Figure S2: 0.05HP@PCL using four different models to evaluate the release profile and mechanism.
