# Supplementary material for: Electrospun Heparin-Loaded PCL Nanofiber Sutures with Sustained Release for Antithrombotic Applications
Source: Pharmaceuticals (Basel). 2026 Sep 2;19(9):1389. doi: 10.3390/ph19091389 (PMC13610262; doi:10.3390/ph19091389)
Supplement: Supplementary file 1 [file pharmaceuticals-19-01389-s001.zip › pharmaceuticals-4520563-supplementary.pdf]

Supplementary Material

1 Supplementary Figures

1.1 Supplementary Figures

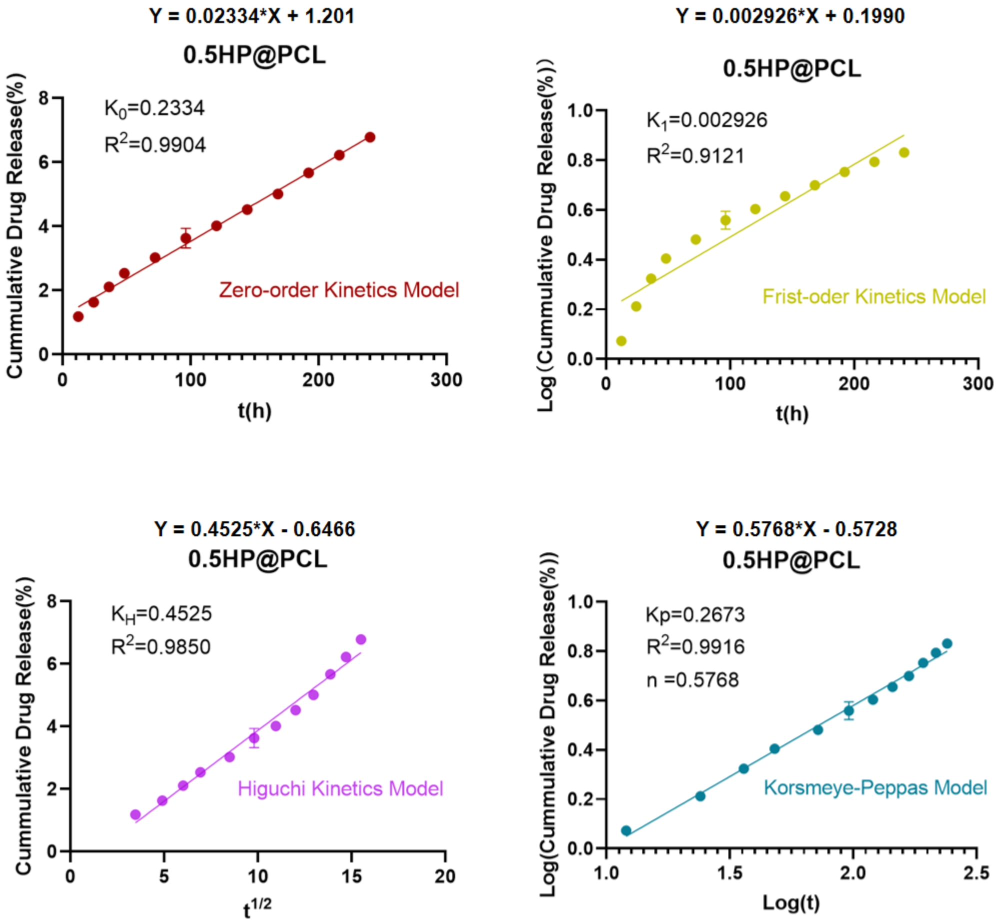

Supplementary Figure S1. 0.5HP@PCL using four different models to evaluate the release profile and mechanism.

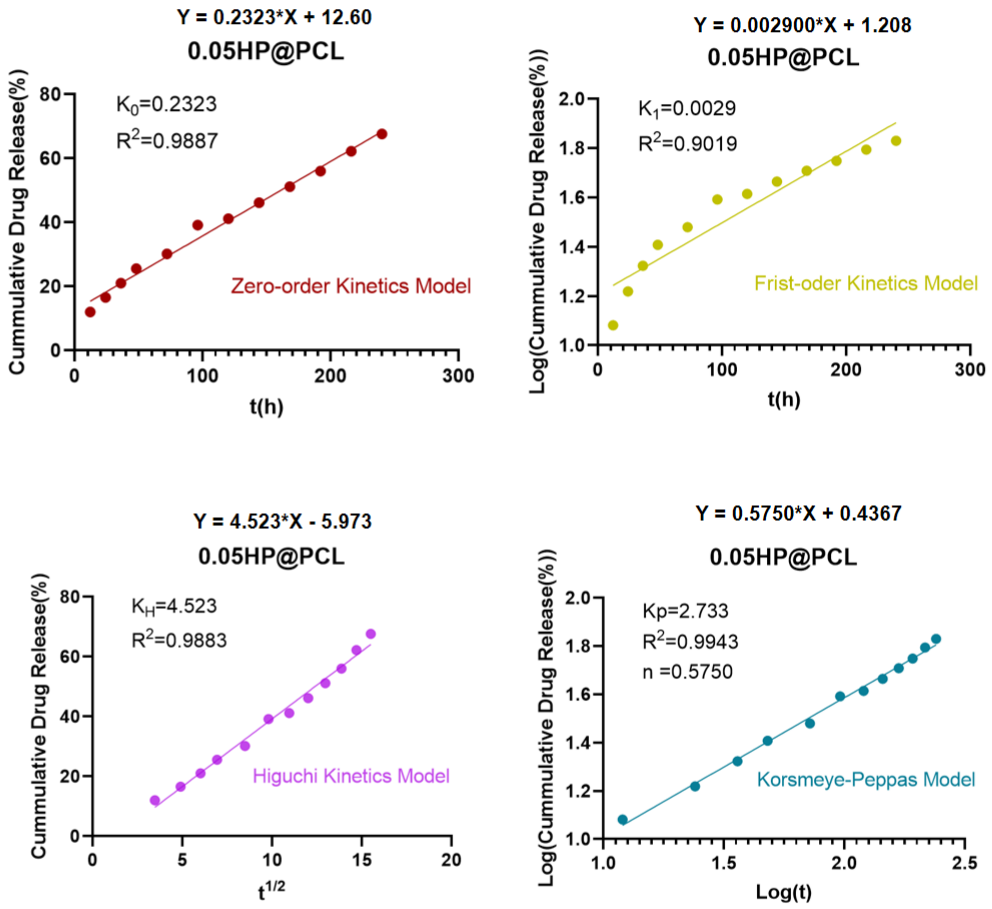

Supplementary Figure S2. 0.05HP@PCL using four different models to evaluate the release profile and mechanism.
